# Patterns of anal carcinoma by gender and marital status in Los Angeles County.

**DOI:** 10.1038/bjc.1983.244

**Published:** 1983-11

**Authors:** R. K. Peters, T. M. Mack

## Abstract

Marital status and other characteristics of 970 residents of Los Angeles County in whom cancer of the anus (including perianal skin) was diagnosed during the period 1972-1981 were compared with those of all county residents and all other persons in whom cancer was diagnosed during the same period. The incidence rate of anal cancer for single males was 6.1 times that for married males (P less than 0.001). This excess was limited to squamous and transitional cell carcinomas and was reasonably consistent by age, stage, subsite, social class and race. Single women were not at increased risk, but separated and divorced persons of both sexes were at increased risk compared to married persons. Anal cancers were more common in males under the age of 35, after which there was a substantial female predominance. This relative excess in older women occurred at all stages, subsites, and social classes of whites and also in blacks, but not in Hispanics, among whom women had lower overall incidence rates compared to both whites and blacks. The findings were consistent with the hypothesis that sexual activity involving the anus is related to anal cancer. We could not rule out the possibility that anal cancer is related to the acquired immune-deficiency syndrome (AIDS) since the incidence in young single men appears to have increased in 1980 and 1981.


*Br. J. Cancer* (1983), **48**, 629–636

### Patterns of anal carcinoma by gender and marital status in Los Angeles County

R.K. Peters & T.M. Mack

*Department of Preventive Medicine, University of Southern California School of Medicine, Los Angeles, California, U.S.A.*

**Summary** Marital status and other characteristics of 970 residents of Los Angeles County in whom cancer of the anus (including perianal skin) was diagnosed during the period 1972–1981 were compared with those of all county residents and all other persons in whom cancer was diagnosed during the same period. The incidence rate of anal cancer for single males was 6.1 times that for married males ($P<0.001$). This excess was limited to squamous and transitional cell carcinomas and was reasonably consistent by age, stage, subsite, social class and race. Single women were not at increased risk, but separated and divorced persons of both sexes were at increased risk compared to married persons. Anal cancers were more common in males under the age of 35, after which there was a substantial female predominance. This relative excess in older women occurred at all stages, subsites, and social classes of whites and also in blacks, but not in Hispanics, among whom women had lower overall incidence rates compared to both whites and blacks. The findings were consistent with the hypothesis that sexual activity involving the anus is related to anal cancer. We could not rule out the possibility that anal cancer is related to the acquired immune-deficiency syndrome (AIDS) since the incidence in young single men appears to have increased in 1980 and 1981.

Recently case reports of anal cancer in male homosexuals (Li *et al.*, 1982; Leach & Ellis, 1981; Cooper *et al.*, 1979) and epidemiological studies showing high risk for anal cancer in single (*versus* married) men (Daling *et al.*, 1982; Austin, 1981 unpublished) have suggested that this cancer may follow habitual anal intercourse. Los Angeles County is a major metropolitan area with a large homosexual population (estimated at $>10^5$). Using a population-based tumour registry covering this county, we have examined the pattern of incidence of this tumour in both men and women by marital status in relation to histological cell type, subsite, stage, age, race, and social class.

### Materials and methods

Ten years of incidence data (1972–1981) from the Los Angeles County Cancer Surveillance Program (CSP) are now available. The methods used by this tumour registry have been described previously (Hisserick *et al.*, 1975; Mack, 1977) and are believed to achieve essentially complete ascertainment of cancer incidence among residents of Los Angeles County. The neoplasms ascertained include surface cancers of the genitalia, anus, and perineum. Cases are identified from hospital and clinic pathology records as well as from death certificates. For each case, address, date of birth, race, sex, marital status, basis for the diagnosis, nature and duration of prior symptoms and other pertinent data are abstracted from the hospital records.

All white cases are classified into Hispanic or non-Hispanic on the basis of surname using the 1970 census surname list (U.S. Bureau of the Census, 1969). Social class is assigned according to the educational and economic characteristics of persons in the census tract of residence at the time of diagnosis (Henderson *et al.*, 1975). Separated and divorced persons are noted separately, but have been grouped together here, since neither group is large and both represent stages in the same decision process.

Age-, sex-, race-, and marital status-specific denominators are available from the 1970 census and are adjusted for undercounting and intercensus change (U.S. Bureau of the Census, 1972; Siegel, 1973 unpublished). Annual age-adjusted incidence rates per $10^5$ (AAIRs) are calculated by direct standardization using ten-year age groups weighted according to the 1970 United States population. An incidence rate ratio (IRR) is a ratio of two AAIRs for mutually exclusive categories and can be thought of as the ratio of incidence in one (exposed) group to that in another standard (unexposed) group. A proportional incidence ratio (PIR) is defined as the ratio of all observed cases of a given cancer in a given category (e.g., married) to

Correspondence: R.K. Peters

Received 6 May 1983; accepted 14 July 1983.

© The Macmillan Press Ltd., 1983

the sum of the age-specific expected cases based on the number of all cancer cases of the same age, sex, and race and the proportion of all such cases of cancer at the site in question. The PIR is thus an indirectly age-adjusted estimate of risk for a given category based on the assumption that persons should experience the same distribution of cancer incidence by site as do others of the same age, sex, and race. A proportional incidence risk ratio (PIRR) is a ratio of two PIRs for two mutually exclusive categories (e.g., single *versus* married). Here we report both IRRs and PIRRs for each association examined because each redresses the limitations of the other. For example, the proportional incidence method relies on the assumption that the incidence of cancer at other sites in single white men is otherwise identical to that in all white men of the same age. Alternatively, the directly computed incidence relies on the assumption that the definition of "single" is the same when applied to white men by a hospital as it is when applied by the census bureau. Summary Chi Square tests (Mantel & Haenszel, 1959) were used to measure statistical significance.

### Results

Over the 10-year period, 369 males and 601 females with malignant tumours of the anus or perianal skin were identified, yielding AAIRs of 1.2 and 1.6 for white males and females respectively. The majority of these tumours had a squamous (63%) or transitional (23%) cell origin. Other histological categories representing at least 1% of the tumours included adenocarcinomas (7%), Paget's disease (2%), basal cell carcinomas (2%), and melanomas (2%).

AAIRs for single and married white men respectively were 4.8 and 0.8, yielding an overall relative risk (IRR) of 6.1 ($P<0.001$) for single *versus* married men. Comparable AAIRs for single and married white women were 1.3 and 1.4, with an IRR of 0.9 ($P<0.50$).

The excess risk for single men was limited to tumours with a squamous or transitional cell morphology (Table I); the risk for transitional cell (cloacogenic) carcinoma was somewhat greater than for squamous cell carcinoma. Squamous and transitional cell carcinomas were combined for all

**Table I** Age-adjusted incidence rates/$10^5$, relative risks (and frequencies) for marital status, and female-to-male ratios for anal and rectal cancers by sex and histological type. Non-Hispanic whites, Los Angeles County, 1972–1981

| | | *Males* | | | | *Females* | | | | *Female-to-Male Ratio* |
|---|---|---|---|---|---|---|---|---|---|---|
| | | *All* | *Single* | *Married** | *Sep/Div* | *All* | *Single* | *Married** | *Sep/Div* | |
| Anus | | | | | | | | | | |
| Squamous Cell | AAIR | 0.8 | | | | 1.1 | | | | 1.4 |
| | IRR | | 6.6† | 1.0 | 1.7 | | 1.2 | 1.0 | 2.0† | |
| | PIRR | | 4.2† | 1.0 | 1.5 | | 1.0 | 1.0 | 2.0† | |
| | (f) | | (63) | (90) | (18) | | (20) | (137) | (55) | |
| Transitional Cell | AAIR | 0.2 | | | | 0.4 | | | | 1.8 |
| | IRR | | 9.1† | 1.0 | 1.4 | | 0.5 | 1.0 | 1.5 | |
| | PIRR | | 8.6† | 1.0 | 1.4 | | 0.6 | 1.0 | 1.6 | |
| | (f) | | (22) | (25) | (4) | | (5) | (64) | (21) | |
| Adeno-carcinoma | AAIR | 0.1 | | | | 0.04 | | | | 0.3 |
| | IRR | | 1.4 | 1.0 | — | | — | 1.0 | 0.6 | |
| | PIRR | | 1.3 | 1.0 | — | | — | 1.0 | 0.7 | |
| | (f) | | (3) | (21) | (0) | | (0) | (7) | (1) | |
| All other primary tumours | AAIR | 0.1 | | | | 0.1 | | | | 1.3 |
| | IRR | | 1.4 | 1.0 | 1.6 | | 0.1 | 1.0 | 0.9 | |
| | PIRR | | 1.0 | 1.0 | 0.9 | | 1.1 | 1.0 | 1.1 | |
| | (f) | | (1) | (9) | (1) | | (2) | (8) | (2) | |
| Rectum | | | | | | | | | | |
| Adeno-carcinoma | AAIR | 15.9 | | | | 9.8 | | | | 0.6 |
| | IRR | | 1.1 | 1.0 | 0.9 | | 1.0 | 1.0 | 0.8 | |
| | PIRR | | 1.1 | 1.0 | 0.9 | | 0.9 | 1.0 | 0.9 | |
| | (f) | | (274) | (2613) | (234) | | (202) | (1333) | (256) | |

*Reference standard for IRRs and PIRRs.
†$P<0.01$.

**Figure 1** Squamous and transitional cell carcinoma of the anus. Average annual age-specific incidence rate by marital status for men and women. Whites, Los Angeles County, 1972–1981. --- single; —— married; —●—●— separated/divorced.

subsequent analyses; other morphologies are not considered further.

The excess risk for single men was present at all ages (Figure 1), stages of diagnosis (*in situ* or invasive), and subsites of the anus, as well as within all races and social classes (Table II). A parallel excess risk for separated/divorced white men, usually less than 2-fold in magnitude, was observed to be reasonably consistent over strata of age, stage, subsite, and social class among white and black men. Separated/divorced Hispanic men appeared to be at relatively high risk, more comparable in magnitude to that of single than of separated/divorced non-Hispanic men.

Women in Los Angeles County are at higher risk for anal cancer than men, and this was also true especially for squamous and transitional cell tumours (Table I). In whites, the overall sex ratio (F/M) was 1.5. The female excess was present for all stages and subsites, within all social classes (Table III), and at all ages after 35. Before 35, there was a slight male preponderance (Figure 2). Among blacks, the female excess was greater than among whites; and among Hispanics, it was absent.

There was no consistent excess risk for single women as there was for single men (Table IV). Separated and divorced women, like men, showed a reasonably consistent elevation in risk for anal cancer, relative to the married. This excess prevailed in all strata of stage, subsite, age and race; among whites it was especially strong among the lowest income groups.

Since late 1979, a syndrome of acquired immune deficiency, evidenced by opportunistic infections and Kaposi's Sarcoma, has appeared among young homosexual men, blood recipients, and other ill-defined groups (CDC, 1982; Gottlieb *et al.*, 1981). Among men under 45 in Los Angeles County, 18 cases of anal cancer were observed in 1980–1981, whereas 8.3 cases would have been expected on the basis of previous incidence. This recent excess was greater for invasive (11 *versus* 3.5) than for *in situ*

**Table II** Relative risks (and frequencies) of anal carcinoma* for marital status by stage, subsite, social class and race. Males†, Los Angeles County, 1972–1981

| | | *Single* RR | (*f*) | *Married‡* RR | (*f*) | *Sep/Div* RR | (*f*) |
|---|---|---|---|---|---|---|---|
| Stage | | | | | | | |
| In situ | IRR | 10.5§ | (23) | 1.0 | (17) | 2.5 | (5) |
| | PIRR | 4.4§ | | 1.0 | | 1.9 | |
| Invasive | IRR | 6.8§ | (62) | 1.0 | (98) | 1.5 | (17) |
| | PIRR | 5.4§ | | 1.0 | | 1.4 | |
| Subsite | | | | | | | |
| Perianal skin | IRR | 6.3§ | (17) | 1.0 | (21) | 0.6 | (2) |
| | PIRR | 3.8§ | | 1.0 | | 0.7 | |
| Anal canal | IRR | 6.4§ | (18) | 1.0 | (30) | 2.4 | (8) |
| | PIRR | 3.8§ | | 1.0 | | 2.1 | |
| Anorectum | IRR | 8.9§ | (34) | 1.0 | (40) | 1.7 | (7) |
| | PIRR | 7.7§ | | 1.0 | | 1.7 | |
| Anus Nos | IRR | 6.1§ | (16) | 1.0 | (24) | 2.0 | (5) |
| | PIRR | 4.0§ | | 1.0 | | 1.6 | |
| Social Class | | | | | | | |
| 1–2 (upper) | IRR | 5.7§ | (22) | 1.0 | (40) | 2.1 | (9) |
| | PIRR | 5.3§ | | 1.0 | | 2.9 | |
| 3 (middle) | IRR | 8.4§ | (26) | 1.0 | (26) | 2.3 | (7) |
| | PIRR | 5.6§ | | 1.0 | | 2.0 | |
| 4–5 (lower) | IRR | 7.7§ | (37) | 1.0 | (49) | 0.9 | (6) |
| | PIRR | 3.9§ | | 1.0 | | 0.6 | |
| Race | | | | | | | |
| Black | IRR | 6.2§ | (11) | 1.0 | (7) | 1.4 | (2) |
| | PIRR | 5.1§ | | 1.0 | | 0.9 | |
| Hispanic | IRR | 4.6¶ | (5) | 1.0 | (8) | 5.3¶ | (4) |
| | PIRR | 3.8 | | 1.0 | | 3.9 | |
| Other White | IRR | 7.2§ | (85) | 1.0 | (115) | 1.6 | (22) |
| | PIRR | 4.8§ | | 1.0 | | 1.5 | |

*Squamous and transitional cell carcinoma only.
†Non-Hispanic white unless otherwise specified.
‡Reference standard for IRRs and PIRRs.
§$P<0.01$.
¶$P<0.05$.

(7 *versus* 4.8) tumours, and it occurred primarily among males who were single (8 *versus* 3.8) or whose marital status was not recorded on their hospital charts (4 *versus* 0.3). All cases were symptomatic and none appeared to have been diagnosed as a result of screening. No similar trend toward increased anal cancer incidence in recent years was evident among women or older men.

### Discussion

We have found that squamous and transitional cell carcinomas of the anus are generally more common in women than men, and that single men (but not women) and separated or divorced persons of both sexes have an excess risk for these tumours. These findings cannot be explained by artifacts or

**Table III** Age-adjusted incidence rates/$10^5$ (and frequencies) and female-to-male ratios for anal carcinoma* by stage, subsite, social class and race. Los Angeles County, 1972–1981

| | *Males†* *AAIR¶* | (*f*) | *Females†* AAIR | (*f*) | *Female-to-Male Ratio* |
|---|---|---|---|---|---|
| Stage | | | | | |
| In situ | 0.2 | (50) | 0.3 | (93) | 1.7 |
| Invasive | 0.8 | (195) | 1.2 | (366) | 1.4 |
| Subsite | | | | | |
| Perianal skin | 0.2 | (45) | 0.3 | (75) | 1.4 |
| Anal Canal | 0.2 | (59) | 0.4 | (118) | 1.6 |
| Anorectum | 0.4 | (90) | 0.6 | (181) | 1.5 |
| Anus, NOS | 0.2 | (51) | 0.3 | (85) | 1.3 |
| Social Class | | | | | |
| 1–2 (upper) | 0.3 | (77) | 0.4 | (121) | 1.3 |
| 3 (middle) | 0.3 | (62) | 0.5 | (147) | 1.8 |
| 4–5 (lower) | 0.4 | (101) | 0.6 | (184) | 1.4 |
| Race | | | | | |
| Black | 0.7 | (21) | 1.4 | (48) | 2.0 |
| Hispanic | 0.8 | (21) | 0.7 | (24) | 1.0 |
| Other White | 1.0 | (245) | 1.5 | (459) | 1.5 |

*Squamous and transitional cell carcinoma only.
†Non-Hispanic whites unless otherwise specified.

**Figure 2** Squamous and transitional cell carcinoma of the anus. Average annual age-specific incidence rates by gender. Whites, Los Angeles County, 1972–1981. —— males; - - - females.

**Table IV** Relative risks (and frequencies) of anal carcinoma* for marital status by stage, subsite, social class and race. Females†, Los Angeles County, 1972–1981

| | | *Single* RR | (*f*) | *Married*‡ RR | (*f*) | *Sep/Div* RR | (*f*) |
|---|---|---|---|---|---|---|---|
| Stage | | | | | | | |
| In situ | IRR¶ | 1.2 | (8) | 1.0 | (51) | 1.9 | (17) |
| | PIRR# | 1.0 | | 1.0 | | 1.6 | |
| Invasive | IRR | 0.9 | (17) | 1.0 | (150) | 1.8§ | (59) |
| | PIRR | 0.8 | | 1.0 | | 1.9§ | |
| Subsite | | | | | | | |
| Perianal skin | IRR | 1.8 | (7) | 1.0 | (36) | 2.1 | (13) |
| | PIRR | 1.5 | | 1.0 | | 1.8 | |
| Anal canal | IRR | 0.8 | (6) | 1.0 | (49) | 1.7 | (19) |
| | PIRR | 0.8 | | 1.0 | | 1.9¶ | |
| Anorectum | IRR | 0.4 | (5) | 1.0 | (79) | 1.9§ | (31) |
| | PIRR | 0.5 | | 1.0 | | 1.9§ | |
| Anus NOS | IRR | 1.3 | (7) | 1.0 | (37) | 2.0 | (13) |
| | PIRR | 1.2 | | 1.0 | | 1.7 | |
| Social Class | | | | | | | |
| 1–2 (upper) | IRR | 0.4 | (3) | 1.0 | (67) | 1.2 | (14) |
| | PIRR | 0.5 | | 1.0 | | 1.5 | |
| 3 (middle) | IRR | 1.3 | (11) | 1.0 | (69) | 1.4 | (19) |
| | PIRR | 0.9 | | 1.0 | | 1.2 | |
| 4–5 (lower) | IRR | 1.2 | (11) | 1.0 | (63) | 3.2§ | (43) |
| | PIRR | 0.9 | | 1.0 | | 2.5§ | |
| Race | | | | | | | |
| Black | IRR | 3.1¶ | (7) | 1.0 | (14) | 1.8 | (10) |
| | PIRR | 1.7 | | 1.0 | | 1.2 | |
| Hispanic | IRR | 0.2 | (2) | 1.0 | (10) | 1.1 | (4) |
| | PIRR | 0.8 | | 1.0 | | 1.7 | |
| Other White | IRR | 0.9 | (25) | 1.0 | (201) | 1.8§ | (76) |
| | PIRR | 0.9 | | 1.0 | | 1.9§ | |

*Squamous and transitional cell carcinoma only.
†Non-Hispanic whites unless otherwise specified.
‡Reference standard for IRRs and PIRRs.
§$P<0.01$.
¶$P<0.05$.

selection biases. Cases of anal cancer are symptomatic at diagnosis, and we cannot postulate differences in physicians' or hospital procedures which would so consistently cut across age, sex, social class, and marital status groups. It is possible that some categories of persons, for example, homosexual males, are more reluctant than others to report their marital status accurately, but such a systematic misclassification could only obscure or decrease a true association with marital status. The associations observed for marital status were generally found using both the IRR and the PIRR, despite the differences between them in the method of estimating what the marital status of cases should be under the null hypothesis. The IRR makes no assumptions about overall cancer incidence by marital status, and the PIRR avoids the assumption that marital status is the same whether measured by the census or a hospital. Our findings are also unlikely to be due to chance, and were consistent across strata of age, stage, subsite, social class and race, despite small numbers within many of these strata.

Austin (1981) reported a 4-fold excess of single persons among male compared to female cases both in San Francisco and in the surrounding 4 counties. A similar excess in the proportion of single persons among male but not female anal cancer cases was noted in pooled incidence data from ten scattered U.S. reporting systems (Daling *et al.*, 1982). Case series of anal cancer generally have reported female-to-male ratios between 1.2 and 3.3 (Grinnell, 1954; Kuehn *et al.*, 1964; Stearns *et al.*, 1980; Singh *et al.*, 1981). An increased risk among separated and divorced women has been consistently reported for cervical cancer, another epithelial carcinoma believed to be linked to sexual behaviour (Hulka, 1982).

Our findings cannot be explained easily by factors unrelated to sexual behaviour. The peculiar epidemiological pattern by gender and marital status is not consistent with occupational exposure to soot, tars, mineral oils, or other substances containing polycyclic aromatic hydrocarbons known to cause scrotal and other epithelial cancers (Woodhouse, 1960; IARC, 1973). Smoking, which may be associated with cervical cancer (Clarke *et al.*, 1982; Buckley *et al.*, 1981), and poor hygiene, which has been linked to penile cancer (Merrin, 1980; Muir & Nectoux, 1979), would be expected to produce the opposite sex ratio and no strong correlation with marital status. Inheritance, drug abuse, exposure to radiation, and dietary habits are likewise not suggested by our findings.

The aetiology of anal cancer is not understood, but this tumour has been linked anecdotally with a prior history of chronic anal conditions such as haemorrhoids, fissures, fistulae, and condylomata acuminata (Grinnell, 1954; Siegel, 1962; Kuehn *et al.*, 1964). In one clinical series, 41% of the cases were found to have been preceded by benign anorectal disease present for at least 5 years prior to diagnosis of the tumour (Buckwalter & Jurayj, 1957); clinically undetected anal carcinomas have been found in one to 2% of tissues removed during routine anorectal surgery for chronic anal conditions (Gordon, 1956; Grodsky, 1967). Although pregnancy is associated with symptomatic haemorrhoids, and thereby might account for some of the female preponderance of anal cancer, we know of no excess of benign anal conditions of non-sexual origin among divorced persons or single men.

Homosexual men are at increased risk for a wide range of benign anal conditions; and a special term—the gay bowel syndrome—has been used to describe a chronic pattern of recurrent anal, rectal, and even colonic diseases which occur with increased frequency among them (Sohn & Ribilotti, 1977; Heller, 1980). These conditions include not only anal warts, gonorrhoea, syphilis and anorectal trauma; but also haemorrhoids, nonspecific proctitis, anal fistula, amoebeiosis, shigellosis, and viral hepatitis. It is assumed that repeated physical irritation and introduction of infectious agents from habitual sexual behaviour involving the anus produce the gay bowel syndrome, and these could also result in anal cancer. Such an explanation is consistent with the recent case reports of squamous and/or cloacogenic carcinomas of the anus in homosexual and bisexual men with histories of habitual anoreceptive anal intercourse (Cooper *et al.*, 1979; Leach & Ellis, 1981; Li *et al.*, 1982).

If sexual behaviours involving the anus increase the risk for anal cancer, an excess risk among single, separated and divorced men would be expected under the assumption that these groups, to varying degrees, are more likely than married men to be homosexual or bisexual, and therefore to practise anal intercourse (Marino & Mancini, 1978). Moreover, the pattern of anal cancer observed among women is probably also consistent with this hypothesis. More women than men are potentially at risk of practising anoreceptive anal intercourse, and separated or divorced women are more likely than married women to have had multiple sexual partners and therefore to have had a partner interested in or willing to engage in sexual behaviour involving the anus. Despite rather strong cultural and even legal taboos, heterosexual anal intercourse is not uncommon (Cornthwaite *et al.*, 1974; Marino & Mancini, 1978; Willcox, 1981; Bolling, 1977). One gynaecologist questioned a consecutive series of 526 patients in 4 different clinic settings in Texas and reported that 25% of the women had experienced anal intercourse at least once, and as many as 8% practised it regularly as a means of achieving pleasure, orgasm, and/or contraception (Bolling, 1977).

An association between anal cancer and anal sexual practices could be mediated by various mechanisms. Mechanical irritation could produce a hyperplastic response. Chemical carcinogens could be contained in anal lubricants and/or cleansers. Infection with an oncogenic virus could be transmitted through digital, oral, or genital contact with the anus. Human sperm and/or smegma have been described as plausible vehicles for oncogenic viruses in cervical carcinogenesis (Alexander, 1973). Both herpesvirus II and papillomavirus (causing condylomata acuminata) are possible candidates by virtue of their association with cervical cancer (Graham *et al.*, 1982; Reid *et al.*, 1982; Hulka, 1982). Condylomata have been described in case reports as apparent precursors to squamous carcinomas of the anus (Shelly & Wood, 1981; Prasad & Abcarian, 1980; Siegel, 1962; Oriel & Whimster, 1971).

No information about the pattern of anal intercourse is available, and several apparent inconsistencies in the overall distribution of anal carcinoma by gender and marital status may reflect either differences in the distribution of the practice of anal intercourse or, since the numbers are small, chance. These include the male preponderance before the age of 35 years, the absence of a female predominance among Hispanics, and the stronger effect of marital status in women, but the weaker effect in men, in persons of lower social class. With respect to the latter finding, the prevalence of both separation and divorce increases directly with decreasing social class among cancer cases in general in Los Angeles and therefore, presumably, in the population. This would result in proportionally more heterosexuals in the pool of separated and divorced men in lower social classes, and thereby a dilution of the excess risk associated with homosexuality. Likewise, lower social class women who are separated/divorced may have a greater mean number of different sexual partners than women in higher social classes where remarriage is more common and comes sooner.

Since the excess risk for anal cancer in single males exists for older as well as younger men and has been observed over at least the last 10 years, and since squamous tumours such as anal carcinoma probably require several years to develop, it seems unlikely that anal cancer is related to AIDS (CDC, 1982; Gottlieb *et al.*, 1981). Nonetheless, we cannot completely rule out this possibility. A small (2- to 3-fold) excess of anal cancer in men under age 45 did occur in the years 1980–1981 compared to the previous 6-year period. Since these excess tumours were symptomatic and predominantly invasive, they cannot be explained by an increase in screening surveillance among these younger and predominantly single men.

Though the present findings seem unlikely to have been produced by non-sexual factors, additional studies are needed to identify the specific behaviour, conditions, and/or infections which produce this tumour. Toward this end, we are presently initiating a case-control study of anal carcinoma. Meanwhile, it may be prudent for physicians to increase their surveillance of anal lesions both in men and women who are known to practise anal intercourse.

The authors are indebted to Diane Kerford and Margaret F. Miller for their assistance in analyzing this data and typing this manuscript.

Supported by grants from the National Cancer Institute: PO-1 CA17054 and the National Cancer Society: #SIG-1.

**References**

ALEXANDER, E.R. (1973). Possible etiologies of cancer of the cervix other than herpes virus. *Cancer Res.*, **33**, 1485.

AUSTIN, D.F. (1981). Etiologic clues from descriptive epidemiology: squamous carcinoma of the rectum or anus. *Paper presented at 3rd Symposium on Epidemiology and Cancer Registries in the Pacific Basin, Maui, Hawaii.*

BOLLING, D.R., Jr. (1977). Prevalence, goals and complications of heterosexual anal intercourse in a gynecologic population. *J. Reprod. Med.*, **19**, 120.

BUCKLEY, J.D., HARRIS, R.W.C., DOLL, R., VESSEY, M.P. & WILLIAMS, P.T. (1981). Case-control study of the husbands of women with dysplasia or carcinoma of the cervix uteri. *Lancet*, **ii**, 1010.

BUCKWALTER, J.A. & JURAYJ, M.N. (1957). Relationship of chronic anorectal disease to carcinoma. *Arch. Surg.*, **75**, 352.

CENTERS FOR DISEASE CONTROL (CDC) TASK FORCE ON KAPOSI'S SARCOMA AND OPPORTUNISTIC INFECTIONS. (1982). Special report: Epidemiological aspects of the current outbreak of Kaposi's sarcoma and opportunistic infections. *N. Engl. J. Med.*, **306**, 248.

CLARKE, E.A., MORGAN, R.W. & NEWMAN, A.M. (1982). Smoking as a risk factor in cancer of the cervix: additional evidence from a case-control study. *Am. J. Epidemiol.*, **115**, 59.

COOPER, H.S., PATCHEFSKY, A.S. & MARKS, G. (1979). Cloacogenic carcinoma of the anorectum in homosexual men: an observation of four cases. *Dis. Colon Rectum*, **22**, 557.

CORNTHWAITE, S.A., SAVAGE, W.D. & WILLCOX, R.R. (1974). Oral and rectal coitus amongst female gonorrhea contacts in London. *Br. J. Clin. Pract.*, **28**, 305.

DALING, J.R., WEISS, N.S., KLOPFENSTEIN, L.L., COCHRAN, L.E., CHOW, W.H. & DAIFUKU, R. (1982). Correlates of homosexual behavior and the incidence of anal cancer. *J.A.M.A.*, **247**, 1988.

GORDON, B.S. (1956). Unsuspected lesions on anal tissue removed for minor conditions. *Arch. Surg.*, **73**, 741.

GOTTLIEB, M.S., SCHOOFF, R., SCHANKER, H.M. & 4 others. (1981). Pneumocystis carinii pneumonia and mucosal candidiasis in previously healthy homosexual men: evidence of a new acquired cellular immunodeficiency. *N. Engl. J. Med.*, **305**, 1425.

GRAHAM, S., RAWLS, W., SWANSON, M. & MCCURTIS, J. (1982). Sex partners and herpes simplex virus type 2 in the epidemiology of cancer of the cervix. *Am. J. Epidemiol.*, **115**, 729.

GRINNELL, R.S. (1954). An analysis of 49 cases of squamous cell carcinoma of the anus. *Surg. Gynecol. Obstet.*, **98**, 29.

GRODSKY, L. (1967). Unsuspected anal cancer discovered after minor anorectal surgery. *Dis. Colon Rectum*, **10**, 471.

HELLER, M. (1980). The gay bowel syndrome: a common problem of homosexual patients in the emergency department (Abstract). *Ann. Emerg. Med.*, **9**, 487.

HENDERSON, B.E., GORDON, R.J., MENCK, H., SOOHOO, J., MARTIN, S.P. & PIKE, M.C. (1975). Lung cancer and air pollution in South Central Los Angeles County. *Am J. Epidemiol.*, **101**, 477.

HISSERICK, J.C., MARTIN, S.P. & HENDERSON, B.E. (1975). An areawide reporting network. *Publ. Hlth Rept.*, **90**, 15.

HULKA, B. (1982). Risk factors for cervical cancer. *J. Chron. Dis.*, **35**, 3.

INTERNATIONAL AGENCY FOR RESEARCH ON CANCER (IARC). (1973). Certain polycyclic aromatic hydrocarbons and heterocyclic compounds. *IARC Monographs on the Evaluation of Carcinogenicity Risk of Chemicals to Men*, Vol. 3. Lyon, France.

KUEHN, P., BECKETT, R., EISENBERT, H. & REED, J.F. (1964). Epidermoid carcinoma of the perianal skin and anal canal: A review of 157 cases. *N. Engl. J. Med.*, **270**, 614.

LEACH, R.D. & ELLIS, H. (1981). Carcinoma of the rectum in male homosexuals. *J. Roy. Soc. Med.*, **74**, 490.

LI, P., OSBORN, D. & CRONIN, C.M. (1982). Anorectal squamous carcinoma in two homosexual men. *Lancet*, **ii**, 391.

MACK, T.M. (1977). Cancer surveillance program in Los Angeles County. *Natl Cancer Inst. Monog.*, **47**, 99.

MANTEL, N. & HAENSZEL, W. (1959). Statistical aspects of the analysis of data from retrospective studies of disease. *J. Natl Cancer Inst.*, **22**, 719.

MARINO, A.W.M. & MANCINI, H.W.N. (1978). Anal eroticism. *Surg. Clin. N. Am.*, **58**, 513.

MERRIN, C.E. (1980). Cancer of the penis. *Cancer*, **45**, 1973.

MUIR, C.S. & NECTOUX, J. (1979). Epidemiology of cancer of the testis and penis. *Natl Cancer Inst. Monogr.*, **53**, 157.

ORIEL, J.D. & WHIMSTER, I.W. (1971). Carcinoma in situ associated with virus-containing anal warts. *Br. J. Dermatol.*, **84**, 71.

PRASAD, M.L., ABCARIAN, H. (1980). Malignant potential of perianal condyloma acuminatum. *Dis. Colon Rectum*, **23**, 191.

REID, R., STANHOPE, C.R., HERSCHMAN, B.R., BOOTH, E., PHIBBS, G.D. & SMITH, J.P. (1982). Genital warts and cervical cancer. I. Evidence of an association between subclinical papillomavirus infection and cervical malignancy. *Cancer*, **50**, 377.

SHELLY, W.B. & WOOD, M.G. (1981). Transformation of the common wart into squamous cell carcinoma in a patient with primary lymphedema. *Cancer*, **48**, 820.

SIEGEL, A. (1962). Malignant transformation of condyloma acuminatum: Review of the literature and case report. *Am. J. Surg.*, **103**, 613.

SIEGEL, J.S. (1973). Estimates of coverage of the population by sex, race and age in the 1970 census. Annual Meeting, Population Association.

SINGH, R., NIME, F. & MITTELMAN, A. (1981). Malignant epithelial tumors of the anal canal. *Cancer*, **48**, 411.

SOHN, N. & RIBILOTTI, J.G. Jr. (1977). The gay bowel syndrome: Review of colonic and rectal conditions in 200 male homosexuals. *Am. J. Gastroenterol.*, **67**, 478.

STEARNS, M.W., URMACHER, C., STERNBERG, S.S., WOODRUFF, J. & ATTIYCH, F. (1980). Cancer of the anal canal. *Curr. Probl. Cancer*, **4**, 1.

U.S. BUREAU OF THE CENSUS. (1969). *1970 Census General Coding Procedures Manual, Attachments J2.* Washington D.C.: U.S. Government Printing Office.

U.S. BUREAU OF THE CENSUS. (1972). *Public Use Samples of Basic Records for the 1970 Census: Description and Technical Documentation*. Washington D.C.: U.S. Government Printing Office.

WILLCOX, R.R. (1981). The rectum as viewed by the venerologist. *Br. J. Vener. Dis.*, **57**, 1.

WOODHOUSE, D.L. (1960). Environmental skin cancer with special reference to mineral oil carcinogenesis. In *Progress in the Biological Sciences in Relation to Dermatology* (Ed. Rook). London: Cambridge University Press.


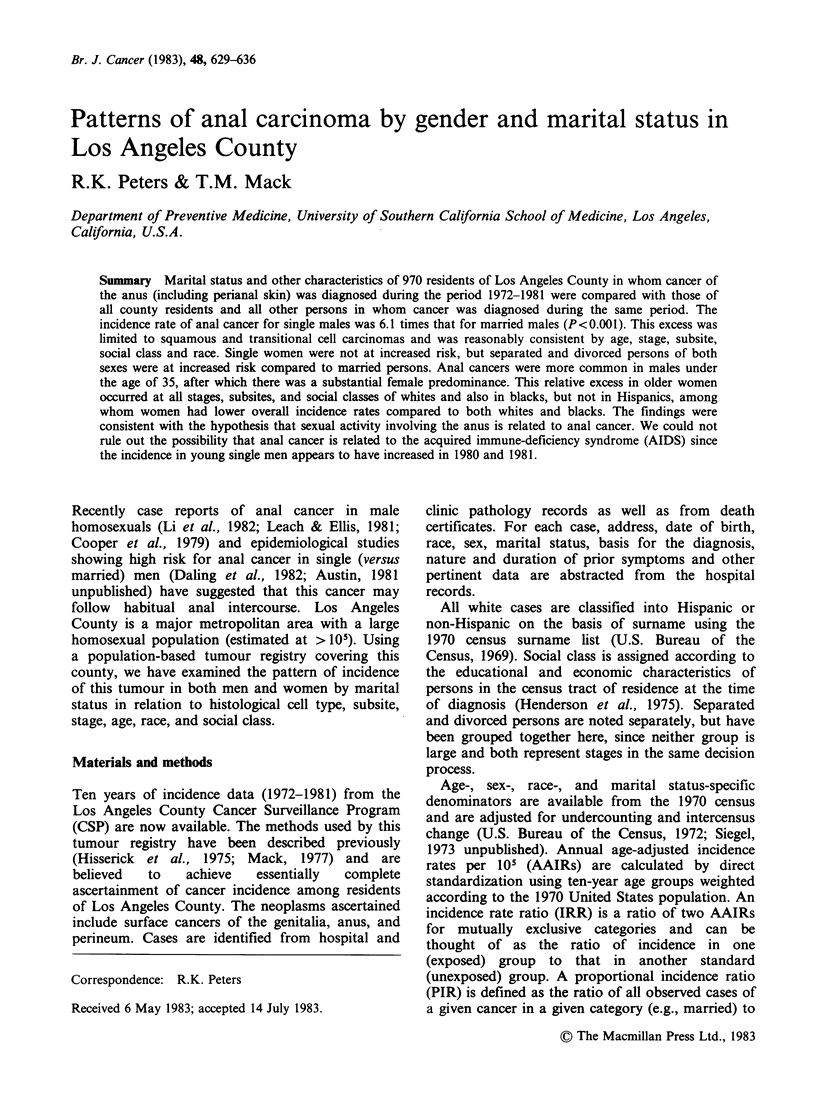

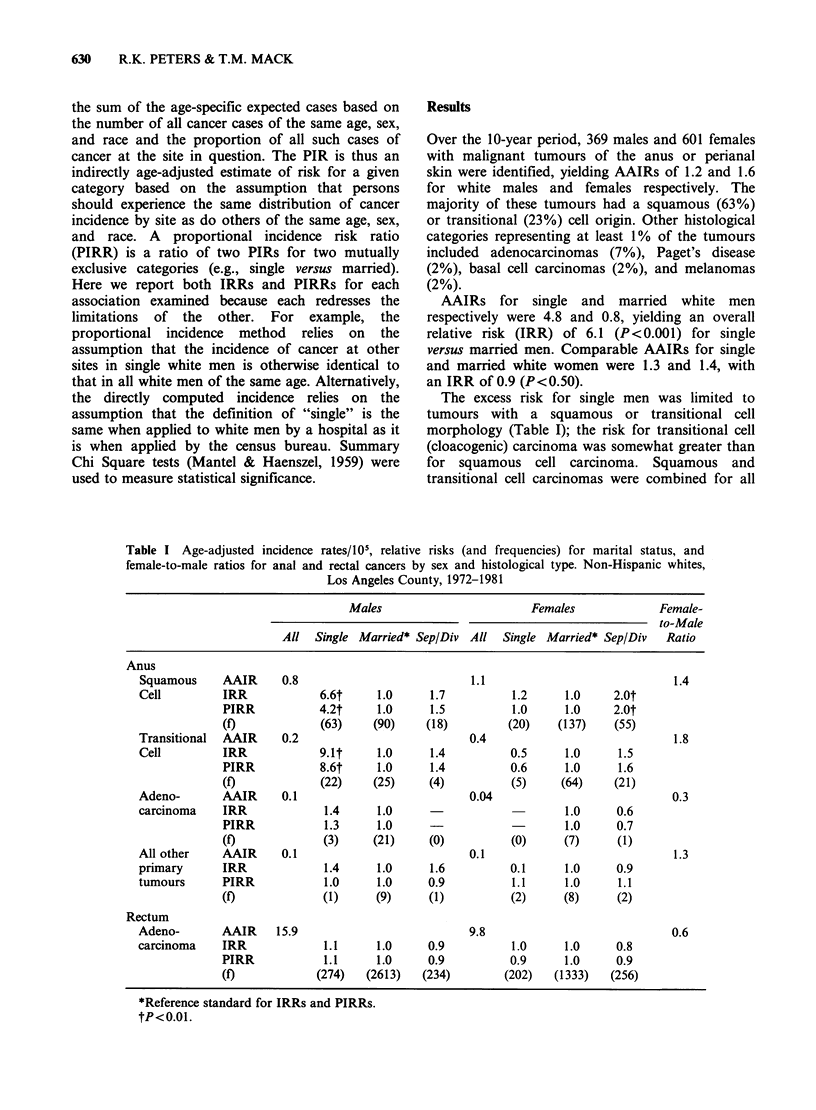

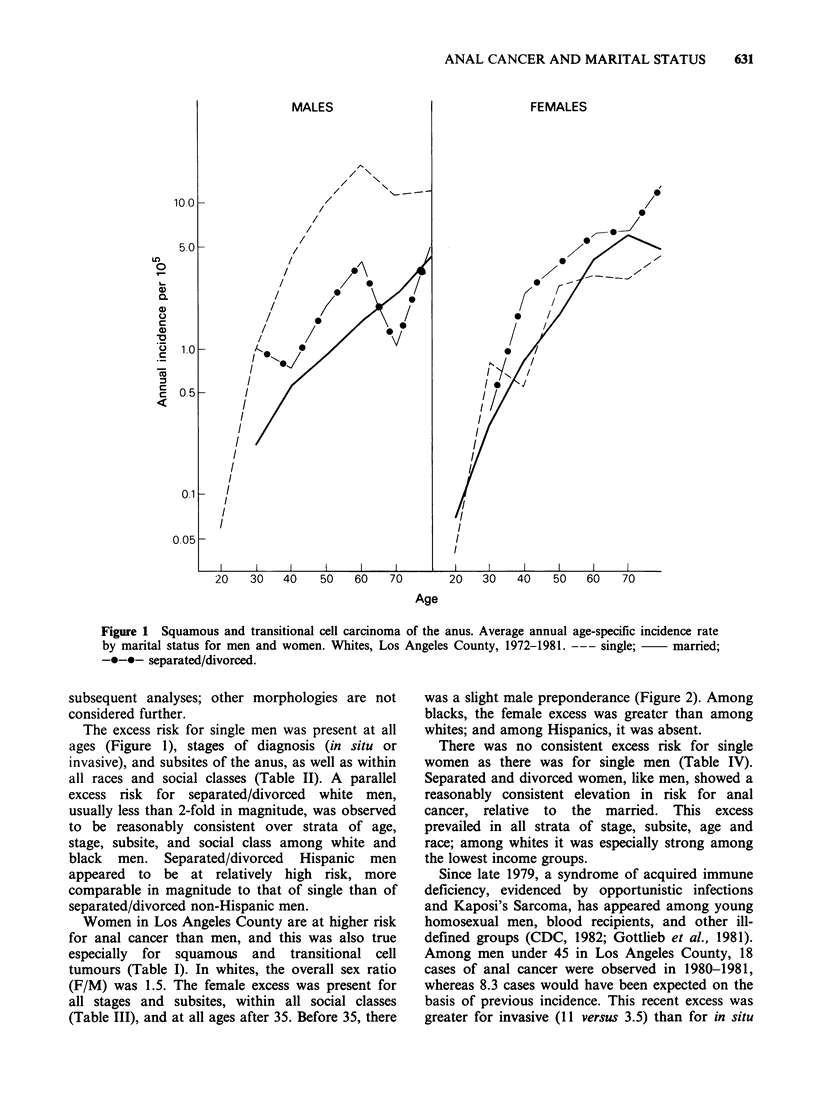

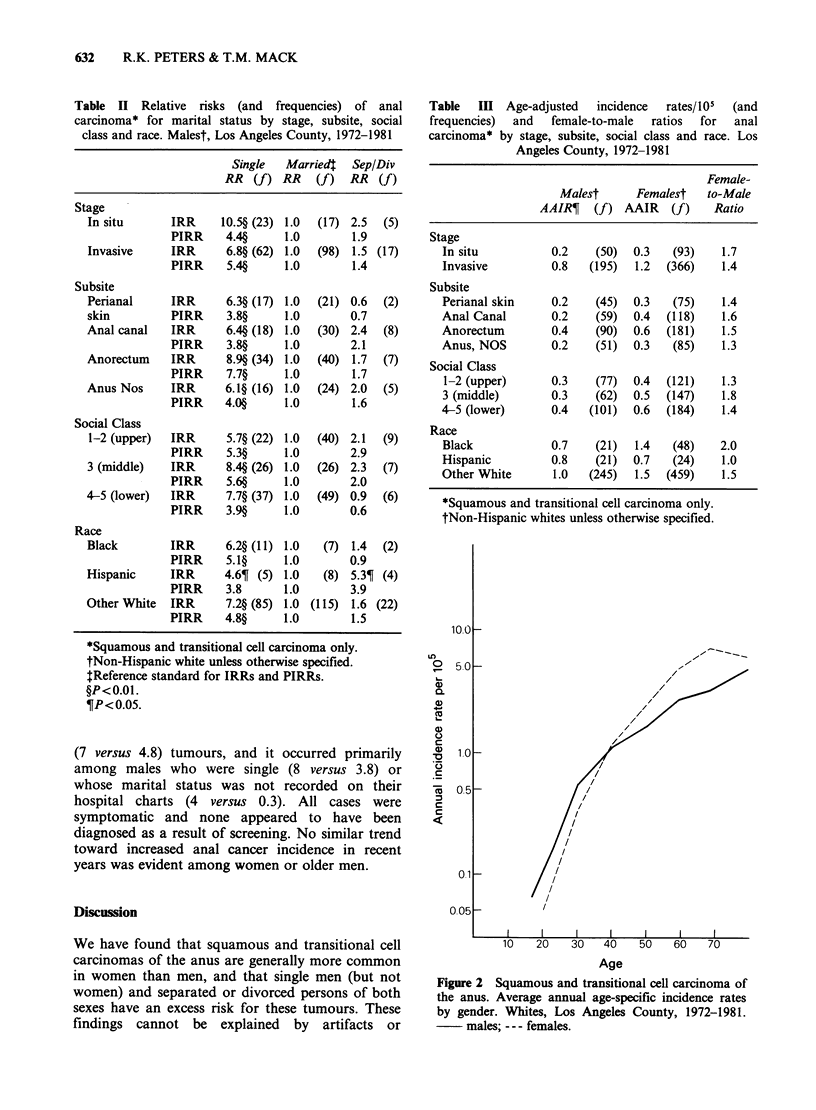

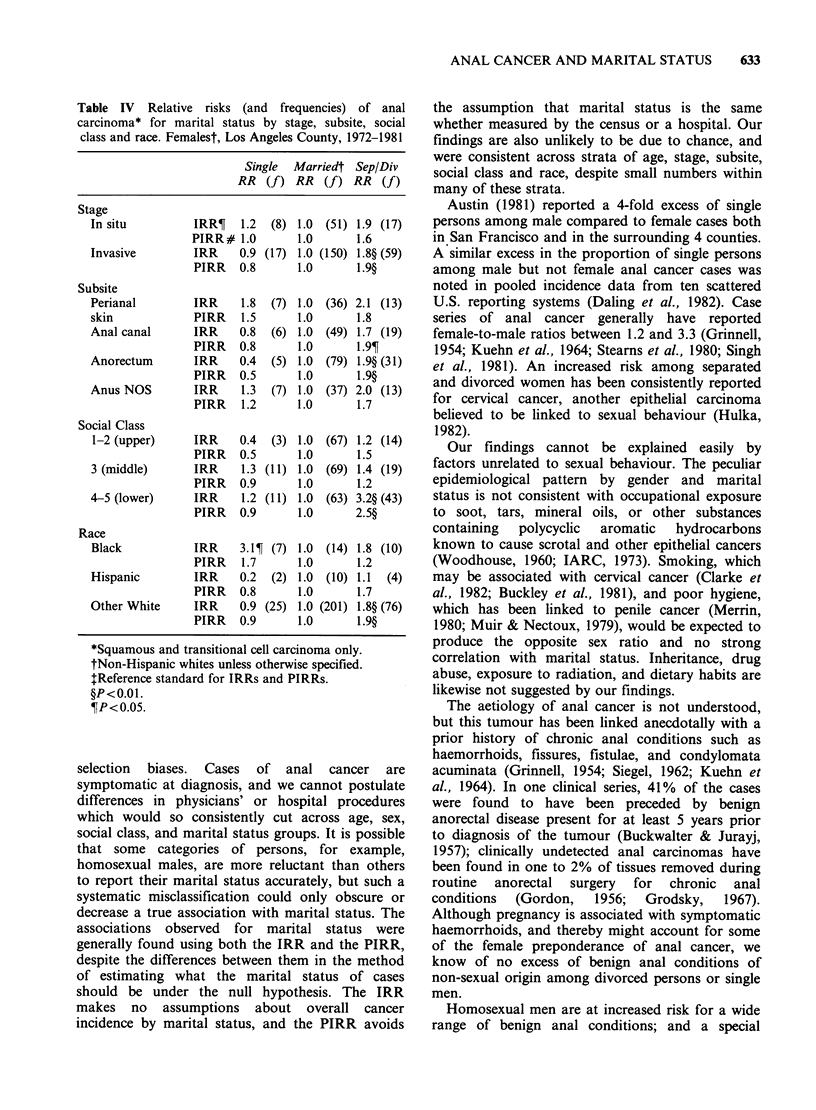

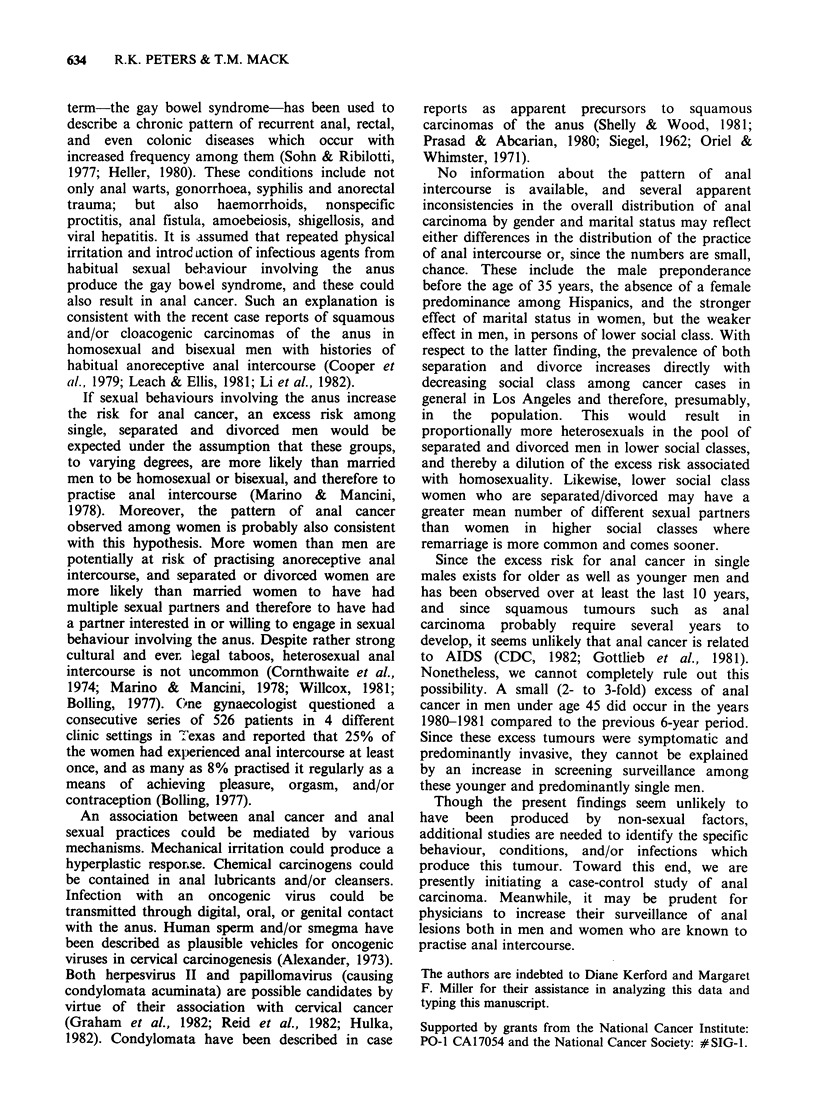

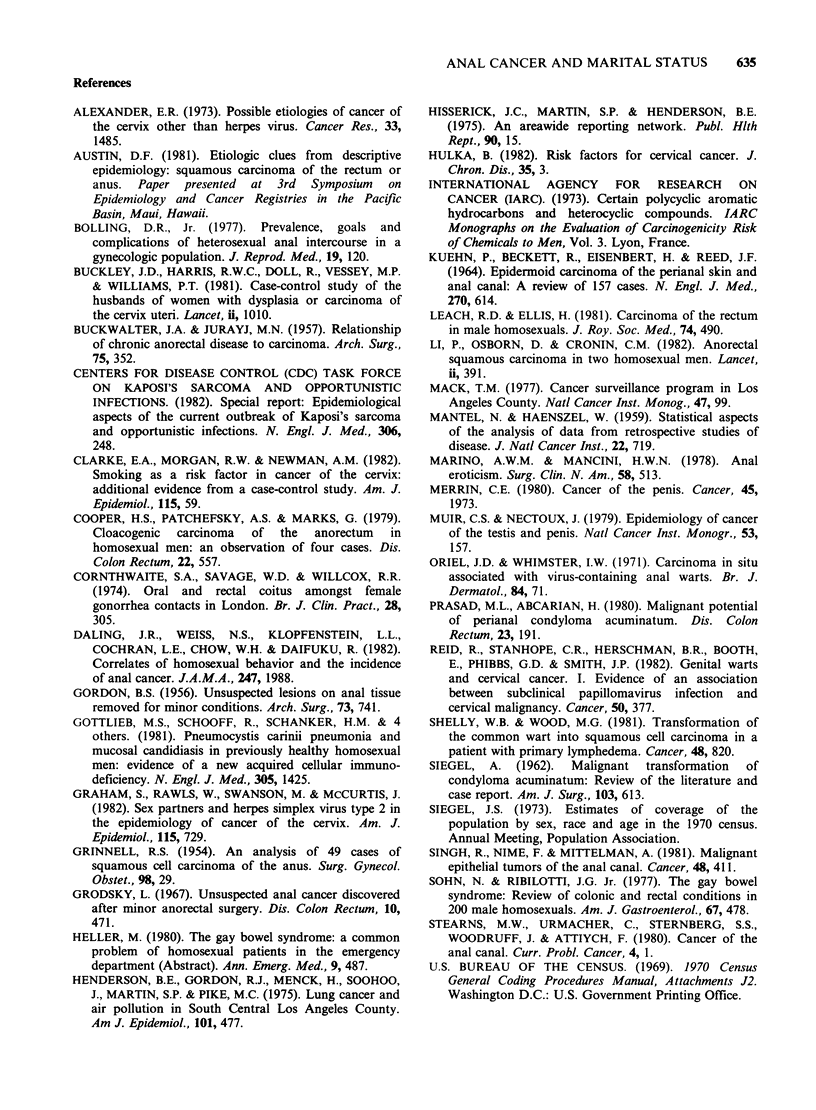

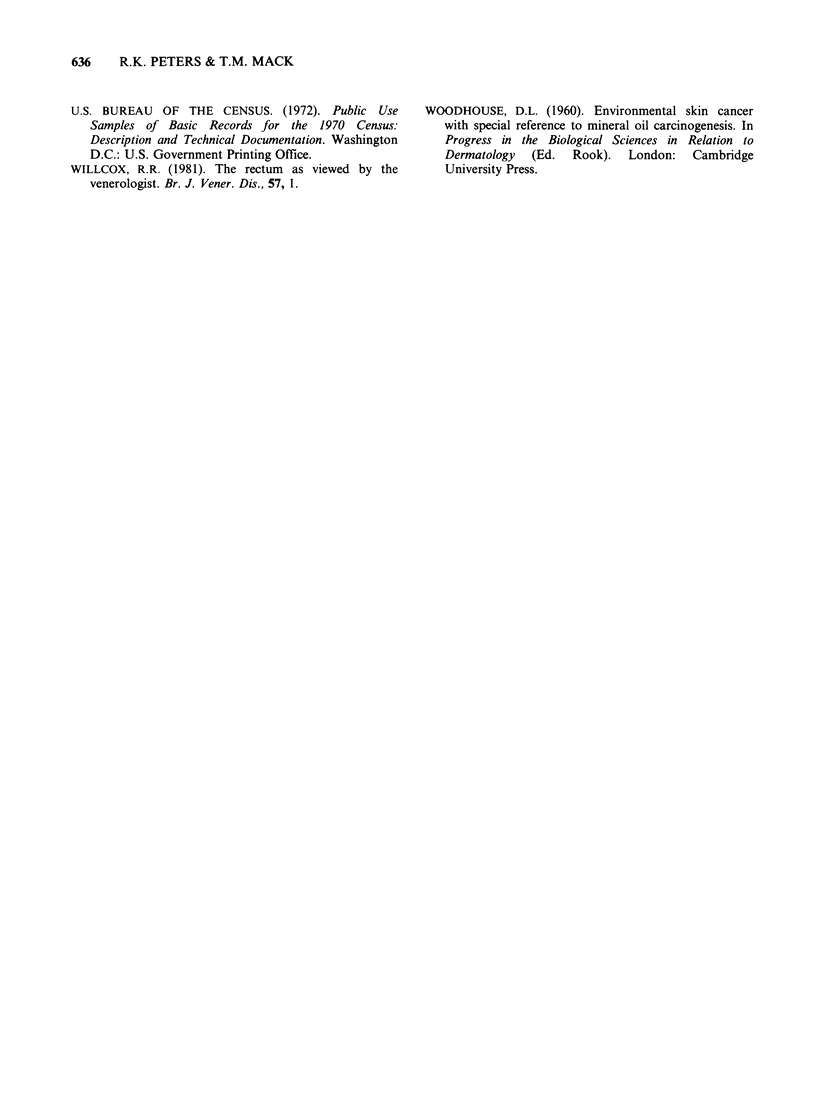


## References

[OCR_00745] Alexander E. R. (1973). Possible etiologies of cancer of the cervix other than herpesvirus.. Cancer Res.

[OCR_00768] BUCKWALTER J. A., JURAYJ M. N. (1957). Relationship of chronic anorectal disease to carcinoma.. AMA Arch Surg.

[OCR_00757] Bolling D. R. (1977). Prevalence, goals and complications of heterosexual anal intercourse in a gynecologic population.. J Reprod Med.

[OCR_00762] Buckley J. D., Harris R. W., Doll R., Vessey M. P., Williams P. T. (1981). Case-control study of the husbands of women with dysplasia or carcinoma of the cervix uteri.. Lancet.

[OCR_00781] Clarke E. A., Morgan R. W., Newman A. M. (1982). Smoking as a risk factor in cancer of the cervix: additional evidence from a case-control study.. Am J Epidemiol.

[OCR_00787] Cooper H. S., Patchefsky A. S., Marks G. (1979). Cloacogenic carcinoma of the anorectum in homosexual men: an observation of four cases.. Dis Colon Rectum.

[OCR_00793] Cornthwaite S. A., Willcox R. R. (1974). Oral and rectal coitus amongst female gonorrhoea contacts in London.. Br J Clin Pract.

[OCR_00799] Daling J. R., Weiss N. S., Klopfenstein L. L., Cochran L. E., Chow W. H., Daifuku R. (1982). Correlates of homosexual behavior and the incidence of anal cancer.. JAMA.

[OCR_00805] GORDON B. S. (1956). Unsuspected lesions in anal tissue removed for minor conditions.. AMA Arch Surg.

[OCR_00822] GRINNELL R. S. (1954). An analysis of fortynine cases of squamous cell carcinoma of the anus.. Surg Gynecol Obstet.

[OCR_00809] Gottlieb M. S., Schroff R., Schanker H. M., Weisman J. D., Fan P. T., Wolf R. A., Saxon A. (1981). Pneumocystis carinii pneumonia and mucosal candidiasis in previously healthy homosexual men: evidence of a new acquired cellular immunodeficiency.. N Engl J Med.

[OCR_00816] Graham S., Rawls W., Swanson M., McCurtis J. (1982). Sex partners and herpes simplex virus type 2 in the epidemiology of cancer of the cervix.. Am J Epidemiol.

[OCR_00827] Grodsky L. (1967). Unsuspected anal cancer discovered after minor anorectal surgery.. Dis Colon Rectum.

[OCR_00832] Heller M. (1980). The gay bowel syndrome: a common problem of homosexual patients in the emergency department.. Ann Emerg Med.

[OCR_00837] Henderson B. E., Gordon R. J., Menck H., Soohoo J., Martin S. P., Pike M. C. (1975). Lung cancer and air pollution in southcentral Los Angeles County.. Am J Epidemiol.

[OCR_00843] Hisserich J. C., Martin S. P., Henderson B. E. (1975). An areawide cancer reporting network.. Public Health Rep.

[OCR_00848] Hulka B. S. (1982). Risk factors for cervical cancer.. J Chronic Dis.

[OCR_00859] KUEHN P. G., BECKETT R., EISENBERG H., REED J. F. (1964). EPIDERMOID CARCINOMA OF THE PERIANAL SKIN AND ANAL CANAL. A REVIEW OF 157 CASES.. N Engl J Med.

[OCR_00865] Leach R. D., Ellis H. (1981). Carcinoma of the rectum in male homosexuals.. J R Soc Med.

[OCR_00869] Li F. P., Osborn D., Cronin C. M. (1982). Anorectal squamous carcinoma in two homosexual men.. Lancet.

[OCR_00878] MANTEL N., HAENSZEL W. (1959). Statistical aspects of the analysis of data from retrospective studies of disease.. J Natl Cancer Inst.

[OCR_00874] Mack T. M. (1977). Cancer surveillance program in Los Angeles County.. Natl Cancer Inst Monogr.

[OCR_00883] Marino A. W., Mancini H. W. (1978). Anal eroticism.. Surg Clin North Am.

[OCR_00887] Merrin C. E. (1980). Cancer of the penis.. Cancer.

[OCR_00891] Muir C. S., Nectoux J. (1979). Epidemiology of cancer of the testis and penis.. Natl Cancer Inst Monogr.

[OCR_00896] Oriel J. D., Whimster I. W. (1971). Carcinoma in situ associated with virus-containing anal warts.. Br J Dermatol.

[OCR_00901] Prasad M. L., Abcarian H. (1980). Malignant potential of perianal condyloma acuminatum.. Dis Colon Rectum.

[OCR_00906] Reid R., Stanhope C. R., Herschman B. R., Booth E., Phibbs G. D., Smith J. P. (1982). Genital warts and cervical cancer. I. Evidence of an association between subclinical papillomavirus infection and cervical malignancy.. Cancer.

[OCR_00918] SIEGEL A. (1962). Malignant transformation of condyloma acuminatum. Review of the literature and report of a case.. Am J Surg.

[OCR_00913] Shelley W. B., Wood M. G. (1981). Transformation of the common wart into squamous cell carcinoma in a patient with primary lymphedema.. Cancer.

[OCR_00928] Singh R., Nime F., Mittelman A. (1981). Malignant epithelial tumors of the anal canal.. Cancer.

[OCR_00932] Sohn N., Robilotti J. G. (1977). The gay bowel syndrome. A review of colonic and rectal conditions in 200 male homosexuals.. Am J Gastroenterol.

[OCR_00937] Stearns M. W., Urmacher C., Sternberg S. S., Woodruff J., Attiyeh F. (1980). Cancer of the anal canal.. Curr Probl Cancer.

[OCR_00955] Willcox R. R. (1981). The rectum as viewed by the venereologist.. Br J Vener Dis.

